# Surgical Treatment of Vitiligo

**DOI:** 10.3390/ijerph19084812

**Published:** 2022-04-15

**Authors:** Alicja Frączek, Marta Kasprowicz-Furmańczyk, Waldemar Placek, Agnieszka Owczarczyk-Saczonek

**Affiliations:** 1 School of Medicine, Collegium Medicum, The University of Warmia and Mazury, 10-082 Olsztyn, Poland; 2 Department of Dermatology, Sexually Transmitted Diseases and Clinical Immunology, The University of Warmia and Mazury, Al. Wojska Polskiego 30, 10-229 Olsztyn, Poland; martak03@wp.pl (M.K.-F.); w.placek@wp.pl (W.P.); aganek@wp.pl (A.O.-S.)

**Keywords:** vitiligo, vitiligo surgery, dermatosurgery, tissue grafts, cellular grafts

## Abstract

Vitiligo is described as a dermatological condition characterized by pigmentation disorders in both the skin and mucous membranes. Clinically, this disease is characterized by the presence of well-defined white areas of various shapes and sizes, which are a manifestation of a reduced number of melanocytes. Due to the fact that vitiligo can be a significant cosmetic problem for patients, a number of methods are currently available to help fight for a better skin appearance. If all the available non-invasive procedures turn out to be ineffective, surgery can help, which is a very good alternative in the case of difficult-to-treat but stable changes. Both the development of new techniques and modifications to the already available treatment of cell and tissue transplantation give hope to numerous patients around the world. The effectiveness of a particular method is determined by its appropriate selection depending on the lesions undergoing therapy. Each form of surgical intervention has its advantages and disadvantages, which, along with the location or size of the treated hypopigmentation area, should be analyzed by a doctor and discussed with their patient. This article is an overview of the currently available methods of surgical treatment of vitiligo and a comparison of their pros and cons.

## 1. Introduction

Vitiligo is a hypopigmentation disorder characterized by a reduction of melanocytes—pigment cells from the basal layer of the epidermis—which is expressed by white macules or patches presented both on the skin and mucous membranes. Although the exact etiology of this disease is still unclear, there are several hypotheses that try to explain the cause of depigmented skin changes: nervous, cytotoxic, and autoimmune [1]. Patients diagnosed with vitiligo have an increased risk of developing Addison’s disease, diabetes mellitus, pernicious anemia, and rheumatoid arthritis [2], as well as hidradenitis suppurativa [3] or alopecia areata [4]. While other systemic diseases—in particular, diseases with an immune/autoimmune and inflammatory background—are observed more frequently in vitiligo, this skin disorder is not considered life-threatening. However, the psychological aspect plays a very important role in the lives of people affected by this kind of health complication [5]. It has been proven that patients with vitiligo present severely lowered self-esteem, which, in turn, affects social behavior and may determine the development of depression, whereby this problem is more often observed among women than men [6,7]. Due to the fact that vitiligo is the most common hypopigmentation disease, the prevalence of which is estimated at 0.1–2%, depending on the sources [8], numerous researchers from around the world are trying to improve the available forms of treatment as well as develop new techniques, due to which the therapeutic effect will be able to bring the best possible results. Presently, there are several methods for treating hypopigmentation changes in the course of vitiligo. In the case where commonly used therapies, mainly those based on topical corticosteroids and phototherapy, do not give satisfactory results, surgical treatment should be considered [9,10]. 

## 2. Methods for Seeking References

The paper includes data from an overview of literature. Evaluated articles were identified by searching PubMed using the following phrases: vitiligo and surgical treatment. Other phrases which were used are: vitiligo and Koebner phenomenon. One of the filters used was the initial limitation of the searched publications to the last 10 years, although during the writing older articles were also included in the analysis. The search was conducted until 18 March 2022. In total, 541 articles were found, and 84 of them were analyzed.

## 3. Dermatosurgery

The last few decades have brought the development of both new, innovative methods and numerous modifications of the already existing techniques that are used in the surgical treatment of vitiligo. We can distinguish two main surgical methods of vitiligo treatment: one of them is based on tissue and the other one on cellular grafts (Table 1). The therapeutic method with the use of tissue grafts consists in taking a fragment of the skin from the area of the body with proper pigmentation which is then transferred without additional processing with chemical reagents or enzymes to the previously prepared recipient site. For this purpose, a number of tools can be used, including dermabraders and various types of lasers: fractional CO_2_, ablative CO_2_, or Erbium YAG. In addition, micro-needling and suction blistering can be used [11]. Usually, the donor site is not directly visible or exposed to sunlight; that is why harvested skin is usually taken from the inner side of the thighs, buttocks, back, or postauricular area, if the treatment region is varied in the face [11]. In turn, the specificity of cell transplants results from the necessity of processing them first, so that the cells obtained as a result of the transforming procedure can then be used as a graft material, applied to the affected area. Dermatosurgery is usually thought to be most effective in segmental vitiligo [12]. This is mainly due to the fact that the changes observed in the course of SV are usually stable, and this criterion is considered to be the most important for effective treatment with the use of surgical methods [12,13]. Nevertheless, patients with stable non-segmented vitiligo may also successfully receive such treatment if this method is supplemented with other available therapeutic options [10,14]. For this reason, in order to strengthen the clinical results obtained after the completion of surgical procedures, the whole treatment should be enriched with NB-UVB 311 nm or PUVA phototherapy [15]. Due to the fact that the appropriate qualification of a patient for a given treatment method is one of the most important factors influencing its effectiveness, it was necessary to establish proper inclusion and exclusion criteria that would facilitate this process. Bearing that in mind, in 2021, the British Association of Dermatologists developed detailed guidelines for the treatment of people with vitiligo according to which surgery is one of the potential therapeutical options. However, the authors note that the treatment is not widely available on the National Health Service (NHS) but in a limited number of centers with a specialist interest. This document suggests the possibility of using cellular grafting for patients with a stable, segmental, or non-segmental form of vitiligo in whom the other method did not bring the expected results; however, according to the authors, surgical therapies are weakly recommended intervention [16].

## 4. Tissue Grafts

### 4.1. Mini-Punch Graft (MPG)

Minigrafting as an innovative method was presented for the first time in 1978 by Rafael Falabella, who used a dermabrasion device equipped with dental burrs. These caused abrasions with a diameter of 2 to 3 mm, with a depth of less than 1 mm, and 5 mm apart. It was a modification of the previously performed punch grafting in the treatment of hypopigmentation skin disease [17]. After more than 40 years, this technique is still considered as cost-effective and easy to follow, and it is especially recommended for patients with acral vitiligo or for those whose hypopigmentation problem affects, for example, the nipple, lips, or palms area, i.e., zones characterized by irregular shape, which are considered to respond unsuccessfully to medical therapies [18,19,20]. The method involves collecting tissue, usually from the upper part of the thigh or the gluteal area, i.e., from the hidden regions [21], and is carried out with a punch biopsy tool equipped with a blade, which allows the obtention of a cylindrical core of tissue sample [20], whereby the procurement of the tissue may be manual or motorized [18]. In order to make mini-punch grafting most effective, the tool is placed at the donor site in such a way that the largest achievable number of grafts can be collected from the smallest possible area. Then, the tissue grafts obtained in this way are transferred to the previously prepared donor area. Finally, both the treated site and the region where the graft is obtained are secured with dressings, which are removed after four to seven days. The last stage of treatment includes phototherapy, which has a beneficial effect on the repigmentation process [22]. According to the article published by Mohammad Helalat et al., approximately 90% of the patients who underwent mini-punch grafting demonstrated good to excellent repigmentation within six months [9]. The study published in 2021 shows that the pigmentation process can be accelerated thanks to the use of platelet-rich plasma (PRP). However, despite the fact that PRP contains, among others, βFGF, which affects the activity of melanocytes, its use did not have a significant effect on the final result of the treatment [23]. The most frequently mentioned therapeutic complications that determine the final cosmetic effect include cobblestoning and polka dot, the presence of which significantly influences the esthetics of the treated skin [20]. Modifications introduced over the years to, for example, the method of obtaining tissue for a transplant, allow for the reduction of possible complications. A publication presented by Hirobe et al. shows that, thanks to the use of an electric micro-drill to obtain a graft with a diameter of 0.6–1.0 mm and 1.5–1.8 mm deep, the risk of cobblestoning was minimized, as well as pigment spreading being improved [24]. Another interesting idea which, according to the authors, has a positive influence on the final therapeutic effects is the use of transverse needling after mini-punch grafting. The results of the treatment presented by the researchers suggest that this modification allows one to achieve a better degree of repigmentation in a shorter time and with the use of the lowest cumulative dose of NB-UVB compared to MPG and needling performed separately [25]. Notwithstanding the foregoing, according to the current guidelines published by the British Association of Dermatologists in 2021, there is insufficient evidence to allow a further recommendation of this method in the treatment of vitiligo [16].

### 4.2. Suction Blister Epidermal Grafting (SBEG)

**Figure 1:**
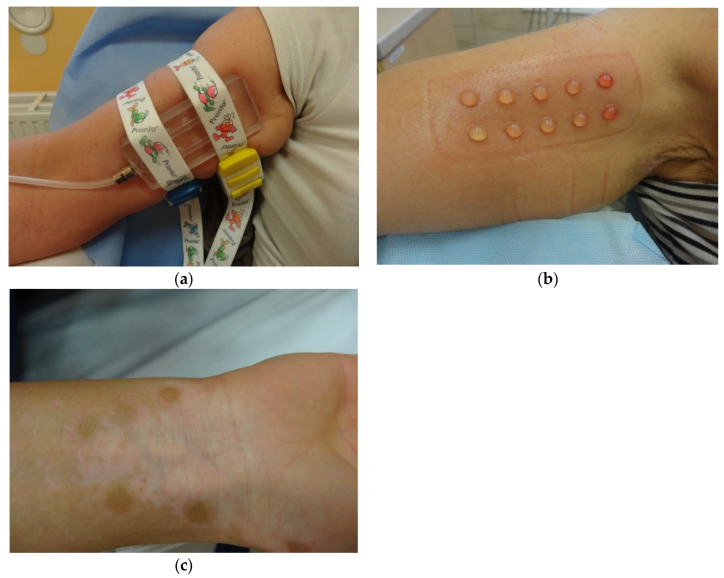
(**a**) Induction of blisters on the forearm by the use of the suction blister-forming dish; (**b**) completed blister formation; (**c**) visible process of repigmentation occurring peripherally from the recipient site.

SBEG as a new method of dermatosurgery was presented for the first time in 1964 by Kiistala and Mustakallio, who decided to separate the epidermis by creating blisters. For this purpose, they used a device called an angiosterrometer, which was originally invented to measure capillary resistance [26]. The idea of SBEG is based on the possibility of collecting the graft by using different suction devices, which, through applying negative pressure, makes it possible to obtain blisters (Figure 1a,b), later used to treat various skin conditions, i.e., hypopigmentation areas in vitiligo (Figure 1c). This method is considered to be easy and safe and can be successfully used around the sensitive area of the mouth and eyelids [11,27]. What is more, there is no need to purchase additional specialized equipment; the blister can be retrieved with a syringe, which from one side—the one without the plunger—adheres directly to the skin and, from the other side, is connected with the needle hub to the suction device, generating negative pressure [28]. Interestingly, the study published by Anabar et al. shows that the rate of blister development depends on the diameter of the syringe used. The authors believe that it is better to use those with a smaller diameter for this purpose [29]. The time required for blister formation is usually from 1.5 to 2 h [27], but due to the nonuniformity of the value and duration of the negative pressure applied, depending on the technique, these parameters may vary. Therefore, the pressure may range from 150 to 500 mmHg [28,30]. What is more, the time needed for generating a blister can be modified by using local anesthetics or increasing the body temperature of the area from which the blister is collected [28,29]. Although the extraction with a syringe seems to be the most convenient way, this method has some drawbacks, including incomplete blister formation and time consumption [31]. For this reason, in recent years, several other techniques have been developed that can be successfully used to obtain suction blisters for grafting [28], such as a special Korean method based on cups with valves [31], modified procedures with syringes including a three-way tap system [32], or a formula with the use of an automated, epidermal graft-harvesting device, called CelluTome^TM^ [33,34,35]. Taking into consideration that scarring after SBEG is a very rare complication [36], all parts of the body seem to be suitable for gaining the blister, but the thigh or the forearm are still the preferred sites. Moreover, there are studies that report the effectiveness of using SBEG in areas such as the angles of the lips that are considered particularly difficult to treat [27]. After a successful grafting, complementary treatment in the form of phototherapy can be applied [36,37].

### 4.3. Split-Thickness Skin Grafting (STSG)

The first reports on the use of the split-thickness skin grafting go back 150 years ago. The method developed by Ollier was originally called the cutaneous-epidermal graft because of the way it was performed, but in the literature it is also known as the ultra-thin skin graft [11] or Ollier-Thiersch graft, due to the similarity of the techniques patented by these two researchers [38]. Another modification of the STSG includes the Blair and Brown method, who, compared to the Ollier-Thiersch procedure, decided to enlarge the transplanted tissue with an additional amount of the dermis layer [38]. Similar to other surgical techniques, STSG is a method in which the site of the graft collection is most often the area of the thighs, buttocks, back, arms, or forearms [39,40]. Depending on the thickness of the tissue collected for transplantation, we can distinguish thin (0.15–0.3 mm), intermediate (0.3–0.45 mm), and thick (0.45–0.6 mm) STSG [40]. For this purpose, a surgical knife or an electric dermatome can be used. It is important that the skin flap obtained in this way is of even thickness along the entire length [41]. Notably, a 2011 study involving 50 patients with stable vitiligo shows that the thickness of the graft used in the treatment was of central importance for the final therapeutic effects. The use of thinner skin flaps, compared to thicker grafts, was associated with, i.e., fewer side effects in the form of scarring or infection [42]. Before harvesting the skin graft, the recipient site is prepared by using a dermabrader, ablation laser, or cryotherapy, which results in the separation of the epidermis from the dermis [30]. Despite the fact that this technique allows the treatment of a relatively large region of hypopigmentation in a time-efficient process, STSG has some drawbacks [11]. One of the biggest disadvantages of this method is the observed insufficient adjustment of both the color and texture of the treated area of the skin [43]. However, some studies suggest that split-thickness skin grafting allows for more satisfactory cosmetic effects than the mini-punch procedure described above, especially when this method is used to treat larger areas [41]. An additional problem that may appear is a slight disturbance of sensation within the recipient area [40]. Moreover, suitable surgical skills are essential for the proper conduct of the procedure.

### 4.4. Epidermal Curettage Technique (ECT)

The curettage technique has been known for over several dozen years, yet it was not initially considered one of the possible forms of tissue grafting in the course of vitiligo [44]. However, the clinical effects associated with the use of this method prompted researchers to further analyze this therapeutic option [45]. The ECT procedure is based on the curettage of the previously anesthetized donor area with a sterile curette. Usually, the sacral region is selected for this purpose, and the activity itself is performed until the Auspitz’s sign occurs. The material obtained in this way is then treated with physiological saline or hyaluronic acid, so that, after mixing, all components form a kind of paste, which is placed on a hypopigmentation area. The recipient area is prepared in a similar way to the donor area, with the difference that the skin obtained in this way is not used for anything in the treatment process, and so it is discarded. The final step is to protect the treated area with a membrane dressing for a period of seven days [44]. A modification of this method is the Jodhpur technique, which involves the use of a 2% antibiotic ointment with mupirocin, which is applied to the donor area, and then dermabrasion is carried out until reaching the upper part of the dermis. This action resulted, among others, in a more economical use of a tissue graft [46], and its first effects were observed after about a month [47]. This method allows for the treatment of an area approximately four times smaller than the recipient area [44] and, therefore, can be successfully used in the case of smaller hypopigmentation areas. One of its main advantages is the ease of implementation and the lack of the need for laboratory equipment, so it is also an affordable technique, which makes it possible to achieve very good therapeutic results. One of the possible complications of ECT is skin hyperpigmentation and secondary infections, which are observed both in the donor and recipient sites. However, these are relatively rare side effects [46].

### 4.5. Smash Grafting

Another surgical method used in the treatment of vitiligo is a technique called smash grafting, which is considered to be a modified form of split-thickness skin grafting. The main difference in smash grafting is that the skin taken from the donor is divided into smaller fragments before it is transferred to the recipient. This procedure does not require any sophisticated devices; donor tissue can be achieved by using a simple sterile razor and prepared with sterile scissors [48]. According to Krishnan et al., a crucial element of the entire treatment is the method of collecting the material; it is important that the skin is obtained by the smallest possible cuts so as to harvest 1–2 cm wide sections [48]. Next, the tissue collected in this way is crushed or cut for 15–20 min to obtain a mushy consistency. Thanks to this procedure, the graft taken from the donor side is up to 10 times smaller than the treated region [21]. Then, the properly treated tissue is placed in a bowl of saline [48]. The tissue prepared in this way is then applied in the area of the affected skin, previously prepared with a dermabrader. This procedure violates the continuity of the epidermis and dermis, leading to the excretion of the exudates containing, i.e., growth factors affecting the regenerative processes of the patient’s skin [49]. Since the graft is in the form of pulp, this method does not require considering the side of the collected graft that is applied to the recipient side. Due to the centralization in the way the pigment spreads, it is important to carefully cover the edges of the hypopigmented area with the prepared material. At the end, in order to avoid complications, appropriate antibiotic therapy is applied. After two weeks, during which no other therapy used in vitiligo is applied, supplementary systemic treatment with psolaren is initiated. The first results are visible after 2–3 weeks [48]. The article published in 2012 by Krishnan et al. shows that all of the participants of the study reached the level of repigmentation of over 90%, with no complications, such as scarring or changes in the skin texture. The final effects were observed about six months after starting the therapy [48]. The ease of performing the procedure, its effectiveness, and low cost of treatment make this method an attractive form of treatment for both patients and dermatologists [50].

### 4.6. Flip-Top Grafting

Flip-top pigment transplantation was, for the first time, presented by Thomas McGovern et al. over 20 years ago. In response to the need for developing newer and more effective therapeutic methods, he decided to create an innovative dermosurgical technique for patients for whom previously available treatments did not bring the expected results [51]. Despite bringing very good clinical results, the method proposed by the researchers requires quite high manual skills. The flip-top method involves creating a special type of skin fold located at a distance of 5–10 mm from the area of the affected skin, under which the tissue harvested with a razor blade is placed. After donor site anesthesia with 1% lidocaine with epinephrine, 2–4 mm of the epidermis with a small amount of the underlying dermis is collected. Then, the material is cut into half-thin fragments on a gauze soaked in sodium chloride. The skin cover is made up of a 5 mm flap of the epidermis, which, with its dermal part, connects with the epidermal part of the graft. Then, the whole is covered with 0.1 mL of cyanoacrylate, after which it is dried, and a polyurethane dressing is placed for a period of one week [51]. The first results of the treatment can be assessed already after 20–25 days [52]. The study conducted by Sharma et al., in which flip-top transplantation (FTT) was compared with punch grafting (PG) in stable vitiligo, shows that in 20 patients both methods turned out to present comparable therapeutic efficacy, with 91–100% of repigmentation in the FTT group achieved by 65% of respondents, in comparison to 50% of those treated with PG. The published results also demonstrate that FTT turns out to be more reasonably priced. Additionally, in the FTT method, the level of pigment spreading was significantly higher, and the complication in the form of cobblestoning occurred over two times less frequently [52]. Despite the promising effects of the flip-top pigment transplantation method, further and more advanced research is needed to support the dermosurgical techniques development for the treatment of vitiligo.

### 4.7. Hair Follicle Graft

The concept of a new technique of vitiligo treatment, which consists in transplanting hair follicles, is based on the fact that they have a reserve function for melanocytes. It has been noticed that in the process of repigmentation in patients with vitiligo there is a significant increase in the number of inactive melanocytes, which accumulate, among others, in the outer sheath of the hair follicle, and due to the processes of division, proliferation, and maturation, they cause a renewed increase in the amount of pigment in the areas of the affected skin [53]. The first reports suggesting the presence of amelanotic melanocytes in hair follicle come from 1959, when Staricco noticed the presence of DOPA-negative cells located in the middle and lower portions of the human follicle, along the outer root sheath [54]. It has been proved that only DOPA-positive melanocytes are present in the epidermis of normal skin. Vitiligo patients, in turn, are found to be completely lacking these cells while retaining their DOPA-negative form in the outer root sheath [53]. Importantly, DOPA-negative cells demonstrate the ability to produce pigment when they are brought into an activated state, e.g., by exposure to ultra-violet rays [55] or abrasion of the epidermis [53]. For this reason, researchers from around the world decided to take advantage of this phenomenon and try to use a hair follicle transplant in the area of hypopigmentation changes in order to obtain repigmentation. In order to obtain hair follicles for transplantation, a small rectangular fragment of skin (approximately 1 cm × 3 cm) should be collected, usually from the occipital or temporal part of the scalp. Then, the hair follicles are grafted into previously formed wells, located at intervals of 3–5 mm in the affected area [56]. An 18G needle, a curve cutting needle, a hair transplant device, or a punch biopsy can be used for this purpose. Finally, both the place from which the hair follicles were collected and the area of the treated skin are covered with dressings, which are removed after about one week. The first symptoms of repigmentation are observed after about two weeks from the absorption of the hair follicles [56]. This method is simple to perform and does not require the use of expensive, specialized equipment, but the repigmentation process may not bring the expected results. In order to improve the therapeutics effects, 10 days after the transplant, additional treatment with tacrolimus, corticosteroids, or photochemiotherapy with psolaren and natural sunlight as the source of ultraviolet A (PUVASol) can be used [56]. An additional risk of this technique is the possibility of foreign body granulomas, especially when a layer of scarring epidermis forms over the hair follicle [57].

## 5. Cellular Grafts

### 5.1. Cultured Melanocyte Graft

Melanin is a naturally occurring pigment in the human body, the synthesis of which, in the skin, takes place with the participation of cells located on the basement membrane of the epidermis, called melanocytes. The presence of any dysfunction, both in the process of melanogenesis and within the melanocytes themselves, manifests itself in the form of pigmentation disorders on the body [58]. The progress in the development of new surgical methods has made it possible to try to use an innovative technique, which is the transplant of cultured melanocytes. This treatment, as in the case of other forms of surgical intervention, is dedicated to patients with stable vitiligo who did not respond positively to other commonly available methods. In order to perform a melanocyte transplant, an unchanged skin fragment should be collected from an area of the body with relatively low exposure—the inner thigh or buttock are best suited. For this purpose, a skin razor blade or a skin grafting knife can be used [58]. It is also possible to collect the skin needed for transplantation from the upper surface of the previously formed blisters. Then, with the help of appropriate enzymes, such as collagenase, dispase, or trypsin, the procedure of separating the epidermis from the dermis is carried out. The selection of an appropriate enzyme is crucial for the effectiveness of the therapeutic process. Based on the analysis of 60 articles, the authors of a review published in 2019 suggest a more favorable effect of trypsin, which allows the isolation of a cleaner and more viable line of melanocytes [58]. In the same year, a study was published in which Li et al. indicated the beneficial effect of long-term trypsinization on the activity and purity of melanocyte colonies that could be used in transplants in patients with vitiligo [59]. The next stage is the isolation and proliferation of melanocytes. Then, a cell culture is raised on a properly selected medium, which, enriched with various factors, leads to obtaining high-purity melanocytes. Before transplantation, the affected area must be properly prepared, for example, by dermabrasion or creating blisters that reveal an area ready for grafting within 1–2 days. Less invasive methods that allow the transfer of the cultured melanocytes to the recipient site include injections. This method brings good clinical results and can be successfully used both in adults and children [58]. Among the side effects, infections and Koebnerisation is mentioned [60].

### 5.2. Cultured Epidermal Graft

The method involving the use of cultured epidermal grafts is similar to the technique of transplanting cultured melanocytes. It was first described by O’Connor et al. over 30 years ago, who noticed the possible use of cultured epithelium in the treatment of severe burns [61]. Currently, this technique is considered one of the surgical forms of treating vitiligo. It consists in taking the skin from the area of correct pigmentation and then subjecting it to the trypsinization process in order to separate the epidermis from the dermis. Then, 24 h after seeding, the cells are transferred to the culture medium, which may be supplemented with serum of the patients [62]. The epidermal membrane produced after a few weeks, covered with collagen on one side, is placed on the recipient site, prepared with the aid of a dermabrader. Finally, the grafted area is protected for two weeks with a layer of Vaseline and sterile gauze [63]. This method allows the treatment of large hypopigmentation areas with the use of small biopsies taken from the donor site. The study by Plott et al. shows that this procedure does not cause scarring. Moreover, despite the fact that it ran with different efficacy, the repigmentation process itself guaranteed a very good color match, which was maintained in the treated patients for at least one year of observation [63]. Possible complications with this method include the likelihood of infections and bleeding and the possible risk of transplant rejection. One of the greatest obstacles to the use of cultured epidermal grafts is the cost associated with carrying out the entire therapy [62,63].

### 5.3. Noncultured Melanocyte-Keratinocyte Suspension

Recently, an increase in the popularity of the method based on the grafting of autologous melanocyte-keratinocyte suspension has been observed. This technique was first proposed by French researchers Gauthier and Surleve-Bazeille, who in 1992 published a procedure using noncultured melanocytes, which, together with keratinocytes present in the transplanted suspension, effectively repigmented the treated changes in patients with vitiligo [64]. Over the last 30 years, the manner of carrying out this method has undergone various modifications, but, in its original form, this procedure consisted in taking a small fragment of the skin from the occipital area, previously anesthetized with 1% lidocaine solution. Then, the harvested graft was placed in 0.25% trypsin solution and subjected to 18 h incubation at 4 °C. As a result, easier separation of the epidermis from the dermis was possible. The epidermis obtained in this way was first immersed in the EDTA solution and then in the salt solution. As a result, it was possible to facilitate the mechanical separation of keratinocytes and melanocytes, which were administered in the form of a suspension to the blisters created with the use of liquid nitrogen placed in the affected areas. Finally, both the recipient and donor sites were dressed, and the treatment effects were monitored during follow-up visits [64]. These activities were designed in such a way that the whole procedure lasted two days; therefore, the aim of subsequent modifications was to improve not only the therapeutic effects themselves but also to shorten the time of the whole technique. This method is considered to be effective and safe, but its success largely depends on the appropriate selection of patients undergoing therapy [46,65]. The fortunateness of treatment is influenced, among others, by the type of vitiligo, as well as the location of hypopigmentation changes. On the other hand, on the basis of the available literature, no relationship between the skin type or gender was noticed [66]. However, the repigmentation process itself is more effective among younger patients (<24 years of age) [65]. What is more, the outcome of treatment can be improved with the additional use of phototherapy [65]. One of the main advantages of autologous melanocyte suspension grafting that outweighs other available surgical methods is the possibility to treat large areas of hypopigmentation in relation to the skin fragment collected for the transplant, although the ratio of donor skin to recipient area has not been clearly defined so far [67]. According to the systematic review published in 2021, the higher the expansion coefficient, the less expressed is the process of repigmentation. After reviewing 31 studies, the authors found that, unlike in cultured melanocyte transplantation (CMT), the clinical effect of the noncultured melanocyte transplantation (NSCT) treatment process was worse when the expansion ratio was 1:10 than when the expansion ratio was 1:3 or 1:5, the phenomenon observed for >50% and >75% repigmentation [67]. The most common complications include incomplete color matching as well as the possibility of scarring and a change in the skin texture. Some publications also report a hypopigmentation, which is observed on the donor site [68,69].

### 5.4. Noncultured Follicular Root Sheath Suspension

Another technique that allows us to obtain melanocytes for skin grafts in patients with vitiligo is a method based on the use of a single-cell suspension acquired from the outer sheath of the hair follicle. This method, described for the first time by Mohanty et al. [70], allows the obtention of material for transplantation with the use of the extracting follicles units (FUE) procedure. The suspension obtained in this way includes not only pigment cells but also melanocyte stem cells, keratinocyte stem cells, and hair follicle stem cells [71,72,73]. For this purpose, the authors collected hair in the anagen phase from the occipital area of the scalp, taking care not to damage the hair follicles, which were then rinsed three times with saline with the addition of antibiotics and antifungal agents. Next, the 1.5 h incubation in EDTA allowed them to obtain a suspension of single cells, which were finally filtered and centrifuged. After being suspended in DNEM and cytological examination, the sediment obtained as a result of these activities was ready for transplantation. The entire procedure takes approximately 2–3 h to complete. The site to be treated was properly prepared for transplantation using a dermabrader and then covered with the obtained suspension of hair follicles. The final stage of activities involved the treatment of the treated area with a collagen dressing; all patients were additionally subjected to supportive treatment [70]. No dressing is required on the donor side [74]. Due to the higher content of CD200^+^ cells, which is a hair follicle bulge stem cells marker [74], this method is considered to be an improved version of the technique proposed by Vanscheidt in 2009 for obtaining melanocytic suspension from “plucked” hair follicles [75]. Treatment with the use of noncultured follicular root sheath suspension is considered to be effective, giving the possibility of achieving very good clinical results, including color match and the absence of scarring [76], provided that the disease is stable for at least one year among the treated patients [70]. Moreover, 15–25 follicular units taken during a single procedure provide a source of 300,000 to two million cells in the form of a suspension that can be used to treat about 20 cm^2^ of the depigmentation area. This makes the method attractive, although it requires high skills, both laboratory and manual [74].

## 6. Discussion

The first attempts at skin grafting occurred over 3500 years ago, dating back to ancient Egypt. The first documented successful skin graft was made by Jaques-Luis Reverdin in the 19th century [38]. In turn, just over 100 years later, an American dermatologist, Norman Orentreich, performed the first successful autograft repigmentation in a patient with leukoderma [77]. These events contributed to the development of dermatosurgery, a related medical field. Currently, there are several surgical methods that are used in patients with vitiligo across the world. The success of treatment is influenced by both the proper selection of the technique for the lesions to be treated and the appropriate consideration of the general advantages and disadvantages associated with a given method. Considering the articles cited in this publication, we collected the most frequently mentioned and specific treatments, their advantages and disadvantages, tabulating them below (Table 2). All things considered, surgical methods of treating hypopigmentation changes resulting from vitiligo are used in a situation where other commonly available therapies have not brought sufficiently good clinical effects. Although their use is associated with some side effects, due to the introduction of further modifications to the already patented techniques, as well as the development of new methods, surgical interventions seem to be a good alternative for patients with a stable form of vitiligo. One of the most frequently mentioned side effects is the Koebner phenomenon, which consists in the appearance of previously unseen changes in the skin where it was damaged [78]. The Koebnorization is observed in diseases such as lichen planus, psoriasis, or vitiligo, where the incidence is estimated at up to 62% [79]. The nature of the changes is equal to that in the underlying disease, which means that in these patients hypopigmentation changes are observed as a result of the triggering agent. Since the Koebner phenomenon is observed due to the action of various injury factors, its emergence may occur after surgical procedures. There are reports of new vitiligo changes in connection with the use of the suction blister method in a 14-year-old patient [80]. Therefore, this is one of the reasons why the right choice of the site from which the transplant is collected and the right choice of technique are so important; in patients who have experienced Koebnerization in the past, it would be advisable to think about a method that would allow treating the largest possible hypopigmentation change using the minimum graft size. However, it should be remembered that the Koebner phenomenon is fluctuating: people who have not observed such tendencies before may become Koebner-positive during their lifetime [78]. For this reason, Koebnerization is often precluding the possibility of surgical treatment in patients with vitiligo [81]. Another factor that should be taken into account in those who are considering surgery is the medications that the patient is taking. From a meta-analysis published last year on the side effects of treatments in people with melanoma, presented data show that vitiligo is one of the most common side results in people treated with checkpoint inhibitors and PD-1 inhibitors. Monotherapy with nivolumab or pembrolizumab was associated with the possibility of developing grade 1 and 2 vitiligo [82]. There are also reports of new hypopigmentation changes in hidradenitis suppurativa patients treated with adalimumab [83]. It is, therefore, important to consider the possible therapeutic effect after the use of surgical treatment with the possible skin side action of the medications taken by the patient at the same time.

It is difficult to clearly determine which of the above-described methods is the best. Nevertheless, the data resulting from the systematic review and meta-analysis published by Hyun Jeong Ju et al. in 2021 show that the technique that achieves a repigmentation degree of >90% among the largest number of treated patients is thin skin grafting. The rate of repigmentation in this group was 72.08%; in turn, in the suction blister grafting group, the rate was 61.68%; cultured epidermal cell suspension (CES), 56.82%; noncultured epidermal cell suspension (NCES), 47.51%; punch grafting 45.76%; and noncultured follicular cell suspension, 36.24% (NCFS) [84]. In order to maximize the expected results, the key factor is the appropriate selection of a given method to the patient, type of vitiligo, and the location of the lesions undergoing treatment. Nonetheless, since vitiligo significantly reduces the quality of life, there remains a need to develop new therapeutic methods and improve available techniques. Although our publication is not a systematic review with a meta-analysis, we decided to summarize the effectiveness of each of the methods described by the original articles included in our publication. The effects are presented in Table 3.

## Figures and Tables

**Table 1:** Classification of surgical methods for the treatment of vitiligo.

Tissue Grafts	Cellular Grafts
Mini-punch graft	Cultured melanocyte graft
Suction blister epidermal graft	Cultured epidermal graft
Split-thickness skin graft	Noncultured epidermal melanocyte suspension
Epidermal curettage technique	
Smash graft	Noncultured follicular root sheath suspension
Flip-top pigment grafting	
Hair follicle graft	

**Table 2:** Comparison of advantages and disadvantages of different surgical methods.

Method	Advantages	Disadvantages	References
Mini-punch graft	Safe and simple technique for patients with stable vitiligo involving only small areas of skin up to 5% of surface area	Infection Cobblestoning Graft displacement	Helalat et al., 2012 [9]
		Insufficient evidence to allow a further recommendation of this method in the treatment of vitiligo	Eleftheriadou et al., 2021 [16]
	Considered to be the easiest, fastest, and least expensive High rate of success Very few preventable or manageable side effects Motorized punches allow harvesting more grafts without exerting pressure	Cobblestoning Variegated appearance Color mismatch Static graft Depigmentation of graft Perigraft halo Graft rejection Hypertrophic scars, Motorized MPG requires some degree of expertise compared Need for sterilization of reusable punches and replacement of motorized punch after every 2000 grafts	Chandrashekar et al., 2014 [18]
Suction blister epidermal graft	Easy Cost-effective	Requires surgical skill Milia formation might occur Pigmentation leading to cosmetic mismatch	Kar et al., 2018 [27]
	Possibility to perform the treatment around delicate areas of the face Easy to use Cost-effective No need of additional equipment	Possible bleeding Scarring Shifts in pigmentation Pain	Angeletti et al., 2019 [28]
	Low cost Absence of scarring Possibility of reusing the donor site No limitations of other tissue grafts (minigrafts, pinch grafts, or thin or ultrathin split-thickness grafts), which often lead to mismatch in texture and color	Time-consuming Not suitable for large areas	Iwanowski et al., 2018 [36]
Split-thickness skin grafting	Better cosmetic matching over larger areas using fewer grafts in comparison to MPG	The possibility of contractures and graft rejection in certain sites, including dorsa of fingers and periungal area SSG requires greater skill than MPG Hypertrophic scarring at the recipient site	Khandapur et al., 2005 [41]
	Effective Good cosmetic results	Color mismatch Milia Infection Perigraft halo Hyperpigmentation of the donor site Slower onset of pigmentation and worse cosmetic outcome in comparision to AMT (autologous melanocyte transfer), although there is no statistically significant difference between STSG and AMT group Scarring Koebnerization Depigmentation	Chopra et al., 2020 [43]
Epidermal curettage technique	Simple Cost-effective Can be performed outside the hospital setting using only basic instruments Promotes diffuse, more homogeneous, and faster repigmentation when compared with the PG technique Allows for the donor area to be reused	The author does not predict any particular disadvantages	Machado et al., 2014 [44]
	Simple More cost-effective than epidermal cell suspension method	Better color matching with surrounding skin and yield of graft in epidermal cell suspension method than in epidermal curettage; the difference was not statistically significant	Tyagi et al., 2021 [46]
Smash graft	Effective and economic Does not require considering the side of the collected graft that is applied to the recipient side Does not need as much donor tissue as punch grafting or split-thickness grafting No sophisticated instruments are required	Possible scarring Oozing Infections Delayed healing Hypopigmentation Time-consuming	Kar et al., 2018 [50]
Flip-top pigment grafting	Simplicity Lack of special equipment, minimal scarring Lack of a recipient site dressing	A significant amount of skill is required It can only be performed over small areas Unsuitable for areas of thickened epidermis, such as the palms and soles	Mohammad et al., 2017 [21]
	FTT is equally effective as PG for treating stable vitiligo In FTT, the graft uptake rate is higher Greater pigment spread, the cost of the procedure is lower than with PG	Cobblestoning Hyperpigmentation Variegated appearance might be noticed	Sharma et al., 2013 [52]
Hair follicle graft	Melanocyte and stem cell reservoir The color match is much more acceptable than that with other methods Minimal postoperative hyperpigmentation in the grafted sites Can be easily applied to a small area of vitiligo Can be performed in the eyelash area or at an angle of the mouth Safe Inexpensive No special equipment is required or a sophisticated operation theater Minimal complications	Intraoperative bleeding Inclusion cyst Reactivation of vitiligo after 3–4 months of the procedure, respectively, however, correlation between the procedure and reactivation could not be conclusively established	Thakur et al., 2015 [56]
	Simple to perform, minimal bleeding	The repigmentation process may not be the most effective The risk of foreign body granuloma formation	Sardi et al., 2001 [57]
Cultured melanocyte graft	Extensive vitiliginous areas can be treated by using a small donor skin effective Might be used for both children and adults	Costly Time-consuming Requires a specialist, fully trained staff, and well-equipped tissue laboratories	Zokaei et al., 2019 [58]
	Simple Effective Requires very little donor skin (usually only one-tenth of the recipient site)	Infection Hyperpigmentation Koebnerization	Pandya et al., 2005 [60]
Cultured epidermal graft	Large achromic areas can be treated in one session using cultured epidermal cells grown from a small biopsy No “cobblestone” appearance Excellent results can be obtained in focal and segmental vitiligo (repigmentation in 80 to 100% of cases)	Temporary hyperpigmentation Long-lasting erythema Transformation of cells into a malignant clone Infections Bleeding Hypertrophic scars High cost of the therapy—the need for special laboratory equipment and the high cost of cell expansion and quality controls	Pianigiani et al., 2006 [62]
	Allows the treatment of large hypopigmentation areas with the use of small biopsies No scarring Good color match Cause minimal discomfort to the patient	The expense of epidermal cell culture. The skill required to grow cells in culture A number of supplies are required that are not commonly found in the dermatologist’s office	Plott et al.,1989 [63]
Noncultured melanocyte-keratinocyte suspension(noncultured epidermal cell suspension)	Long-lasting repigmentation Very good color matching	Incomplete color matching The possibility of scarring and changes in the skin’s texture Hypopigmented halo Erythema Infections	Bassiouny et al., 2018 [66]
	Faster procedure for large areas and higher cell count in comparison to outer root sheath hair follicle suspension method	Delayed healing Hyperpigmentation Scarring	El-Zawahry et al., 2017 [68]
Noncultured follicular root sheath suspension	Relatively simple Tiny scars on scalp involves removal of much less volume of tissue in comparison to scalp biopsy (strip) Quicker healing process in comparison to conventional strip method Donor site dressing is not required after FUE Minimally invasive Good yield of melanocytes, melanocyte stem cells, and other stem cells Excellent pigmentation	Requires high laboratory and manual skills	Gupta et al., 2013 [74]
	Preparation of ORS cell suspension is technically less challenging than preparation of epidermal cell suspension	No side effects were observed at the donor site Initial hyperpigmentation that subsequently faded to match normal skin color	Shah et al., 2016 [76]

**Table 3:** The efficacy of available surgical methods.

Method	Type of the Article	Number of Patients	Definition of Efficacy (%)	Repigmentation	Follow up	References
MPG	Original article	29	Excellent repigmentation (>75%)	Postoperatively 58.6% of patients	12 months	Helalat et al., 2012 [9]
	Original article	10	No definition in the article	86.7% of sites repigmented with excellent cosmetic colour match	6 months	Chandrashekar et al., 2014 [18]
	Original article (an intrapatient comparative prospective interventional single-center open-label study)	17	Excellent repigmentation (>90%)	PRP/MPG/phototherapy: 0% of patients MPG/phototherapy: 0% of patches PRP/MPG/phototherapy: 52.9% of patients MPG/phototherapy: 41.2% of patches PRP—platelet-rich-plasma MPG—mini-punch grafting	8 weeks 20 weeks	Salem et al., 2021 [23]
	Clinical trial report (a comparative prospective study)	20	Excellent cosmetic matching: moderate to excellent (50–100%) The authors did not specify the exact percentage of repigmentation considered as “excellent”	Line 1: 47.4% of lesions Line 2: 33.3% of lesions Line 3: 55.6% of lesions Line 4: 18.2 of lesions line-1—mini-punch grafting, line-2—needling, line-3—combined grafting and needling line-4—control group receiving non-procedural treatment	3 months 6 months	Ragab et al., 2021 [25]
SBEG	Original article (prospective study conducted in patients who presented with angle of lip vitiligo)	112	Complete repigmentation: The authors did not specify the exact percentage of repigmentation considered as “complete”	88.2% of patients 88.5% of patients 93.1% of patients 87.5% of patients 89.5% of patients	3 months 6 months 12 months 18 months 24 months	Kar et al.,2018 [27]
	Original article	10	Complete repigmentation (>90%)	0% of patients 7% of patients	3 months 6 months	Iwanowski et al., 2018 [36]
STSG	Original article	64	Number of lesions showing excellent repigmentation (>75%)	Group 1 (MPG): Face: 50%, Trunk: 88.8% Extremities: 17.6% Group 2 (STSG): Face: 91% Trunk: 100% Extremities: 76.4%	3 months	Khandapur et al., 2005 [41]
	Original article (prospective single-center study)	22	Excellent repigmentation (≥75%)	Group A (STSG): 40% of patches Group B (autologous noncultured melanocyte transfer): 42.5% of patches	6 months	Chopra et al., 2020 [43]
Epideraml curettage tachnique	Original article	20	Excellent repigmentation of lesions (>75%)	Group A (noncultured epidermal cell suspension): 0%,0%,8%,12% of lesions Group B (epidermal curettage): 0%, 0%, 25%, 60% of lesions	2 weeks 4 weeks 8 weeks 12 weeks	Tyagi et al., 2021 [46]
Smash graft			No available data			
Flip-top pigment grafting	Original article	20	Excellent repigmentation (>90%)	PG: 0% of patients FTT: 0% of patients PG: 0% of patients FTT: 0% of patients PG: 50% of patients FTT: 65% of patients PG—punch grafting FTT—flip-top transplantation	1 months 3 months 6 months	Sharma et al., 2013 [52]
Hair follicle graft	Original article (a prospective study)	50	Excellent improvement of the lesions (75–100%)	33.3% of lesions	6 months	Thakur et al., 2015 [56]
Cultured melanocyte graft	Original article	27	Excellent repigmentation (>90%)	Cultured melanocyte technique: 50% of patients AMRCS (autologous melanocyte rich cell suspension): 52.2% of patients	6 months	Pandya et al., 2005 [60]
Cultured epidermal graft	Original article	93	Complete repigmentation The authors did not specify the exact percentage of repigmentation considered as “complete repigmentation”	60% of patients	3 months 6 months 12 months 18 months	Pianigiani et al., 2006 [62]
Noncultured melanocyte-keratinocyte suspension	Original article	20	Excellent repigmentation of lesions (>75%)	Group A (noncultured epidermal cell suspension): 0%, 0%, 8%, 12% of lesions Group B (epidermal curettage): 0%, 0%, 25%, 60% of lesions	2 weeks 4 weeks 8 weeks 12 weeks	Tyagi et al., 2021 [46]
	Original article	12	Repigmentation > 90%	33.3% of patients	8 days 3 weeks 1 months	Gauthier et al., 1992 [64]
	Original article (retrospective review)	2283	Long-term excellent repigmentation (>90%) of the skin lesions	66% of patients—segmental vitiligo 53.5% of patients—undefined vitiligo (focal) 46.5% of patients—non-segmental vitiligo	12–108 months	Zhang et al., 2021 [65]
	Original article (prospective multicenter comparative study)	37	Cases with pigmentation ≥ 75% were considered responders	NCECS (noncultured epidermal cell suspension): 20% of patients * ORSHFS (outer root sheath hair follicle suspension): 33.3% of patients * * assesment of the impact of donor tissue variability on the clinical outcome	18 months	El-Zawahry et al., 2017 [68]
	Original article (retrospective review)	100	Long-term excellent repigmenattion (>90%)	Segmental/focal vitiligo: 58% of patients Nonsegmental vitiligo: 36% of patients Physical leukoderma: 12% of patients	12–72 months	Silpa-Archa et al., 2017 [69]
	Original article (prospetive study)	14	Repigmentation ≥ 90%	57.1% of patients	5–15 months	Mohanty et al., 2011 [70]
Noncultured follicular root sheath suspension	Original article	5	Repigmentation > 90%	60% of patients	6 months	Vanscheidt et al., 2009 [75]

## Data Availability

Not applicable.
